# Changes in Sleep Patterns during Pregnancy and Predictive Factors: A Longitudinal Study in Saudi Women

**DOI:** 10.3390/nu14132633

**Published:** 2022-06-25

**Authors:** Sara Al-Musharaf

**Affiliations:** Department of Community Health Sciences, College of Applied Medical Sciences, King Saud University, Riyadh 11451, Saudi Arabia; salmosharruf@ksu.edu.sa; Tel.: +966-55-424-3033

**Keywords:** sleep, PSQI, poor sleep, short sleep duration, vitamin D, pregnancy trimesters, women

## Abstract

This study aimed to assess sleep patterns during the three trimesters of pregnancy and whether vitamin D concentrations, along with other risk factors, are associated with these alterations. In a longitudinal study, 140 pregnant women (age 18 to 39 years) were followed throughout their first, second, and third trimesters. Sleep was measured using the Pittsburgh Sleep Quality Index (PSQI) at each trimester, along with an assessment of biochemical parameters, including serum vitamin D levels. The information that was collected included anthropometric data, socio-economic status, dietary intake, and physical activity. The PSQI was higher in mid and late pregnancy than in early pregnancy (both *p* = 0.001), and the sleep duration was also higher in late versus early pregnancy. Linear regression analyses revealed independent predictors of deteriorating sleep quality from early to late pregnancy, including low income (B ± SE −0.60 ± 0.26, *p* = 0.03) and low serum vitamin D levels in the second trimester (B ± SE −0.20 ± 0.01, *p* = 0.04). Energy intake and sitting in the second half of pregnancy were positively associated with changes in the PSQI score from the second to third trimesters (B ± SE 0.15 ± 0.07, *p* = 0.048) and (B ± SE 0.01 ± 0.00, *p* = 0.044), respectively. Low socio-economic status, low serum vitamin D levels, greater energy intake, and sitting time were associated with worsening patterns of sleep quality from early to late pregnancy.

## 1. Introduction

Sleep needs vary with age, gender, lifestyle, and other factors, such as work schedules and stress [1,2]. Poor sleep is linked to neuroendocrine, metabolic, and inflammatory changes [3]. Studies have also demonstrated that poor sleep quality and inadequate sleep duration may lead to various health complications [4,5]. Poor sleep is common during pregnancy due to physiological and psychological changes [6,7]. However, the prevalence of altered sleep patterns and disturbances has noticeably increased in recent times, i.e., such patterns and disturbances are now considered an epidemic among the general population [8], especially among pregnant women [9]. According to the National Sleep Foundation in 2017, 78% of women reported more disturbed sleep during pregnancy than at any other time in their lives [10]. A meta-analysis showed that the prevalence of poor sleep quality, defined by a Pittsburgh Sleep Quality Index (PSQI) global score of 5 or above, was approximately 44.5% during pregnancy [11,12]. Furthermore, numerous studies have shown a connection between abnormal sleep patterns and a broad spectrum of adverse pregnancy outcomes, including low birthweight, preterm birth, intrauterine growth retardation, caesarean delivery, gestational hypertension, and gestational diabetes [13,14,15,16], along with a decreased quality of life and higher levels of depressive symptoms [17].

Despite the high prevalence of abnormal and disturbed sleep during pregnancy and the adverse health ramifications, studies evaluating changes in sleep patterns and the associated predictive factors across pregnancy trimesters are scarce. Few prospective cohort studies have analyzed changes in sleep patterns during pregnancy in the same cohort of women [16,18,19,20,21,22], with the majority assessing only two time points [12].

Frequent urination, body aches, and sleep positions are among the most well-known reported causes of poor sleep during pregnancy and often vary depending on the trimester [6,23]. However, a broad range of other factors, such as gestational age [12,23], maternal age [11,16], income [24], parity, and race [18], have also been associated with changes in sleep patterns during pregnancy. Obesity and gestational weight gain (GWG) have also been shown to correlate with worsening sleep quality [19,25,26]. Although biochemical markers, such as abnormal glucose and lipid levels, have been linked to poor sleep quality in the general population [25,27,28], to our knowledge, few studies have investigated the relationship between hyperglycemia and gestational diabetes during pregnancy and changes in patterns of sleep [29,30].

Few studies have examined the role that excess macronutrients [31] or deficiencies in micronutrients [32], such as vitamin D deficiency [33], may play in contributing to poor sleep. Vitamin D deficiency is common during pregnancy both globally [34] and in Saudi Arabia [35] and it has been independently linked to pregnancy-related complications that adversely affect the long-term health of mothers and their infants [13,36].

A recent meta-analysis, in which two of the nine included studies examined pregnant women, found that vitamin D deficiency was associated with a 1.5-times increased chance of women experiencing poor sleep quality, short sleep duration, and sleepiness [37]. To our knowledge, limited studies have evaluated the association between vitamin D levels and sleep patterns in a pregnant population [33,38,39,40]. Such studies were conducted in the second or third trimester and did not provide insights into the presence or type of sleep disturbances in early pregnancy, nor did they evaluate changes in sleep patterns throughout pregnancy. Furthermore, most longitudinal studies did not explore biochemical factors, such as glucose, lipid, and vitamin D levels, nor did they consider levels of physical activity or dietary intake in relation to sleep patterns.

Consequently, this study aims to expand our understanding of the types of poor sleep that are experienced by women over the entire pregnancy and identify the predictive factors that increase the risk of poor sleep. Changes in sleep patterns among Saudi women during the first, second, and third trimester of pregnancy were assessed using the PSQI, and the relationship between sleep alterations and biochemical markers such as vitamin D, lipids, glycated hemoglobin (HbA1c), and glucose levels, as well as socio-demographic factors, such as age, parity, body mass index (BMI), GWG, dietary intake, and physical activity, were investigated. Routinely screening pregnant women for sleep-related problems and the presence of potentially modifiable risk factors may contribute substantially to improvements in maternal and fetal/neonatal health outcomes.

## 2. Materials and Methods

### 2.1. Study Design

Pregnant women who previously participated in an earlier prospective cohort study (E-13-1013) made up 24% (141/578) of the participants. The parent study (*n* = 578) assessed serum vitamin D levels with pregnancy-related complications. Ethical approval was obtained from the Ethics Committees of King Saud University Hospital (KSUH) and the Ministry of Health, Riyadh, Kingdom of Saudi Arabia (KSA) (IRB Approval, 14/4067/IRB). Prior to the commencement of the research, written informed consent was obtained from all participating women. The study was conducted in three hospitals in Riyadh city (King Fahad Medical City Hospital, King Khalid University Hospital, and King Salman Hospital) (latitude: 24°42′ N, 46°43′ E) between March 2014 and December 2017, as detailed previously [35]. The current analysis was a secondary analysis of the sleep data that were collected as part of this protocol.

A follow-up was carried out at three different points: early pregnancy (also referred to as the first trimester; 12 ± 3 weeks); mid-pregnancy (also referred to as the second trimester; 26 ± 4.8 weeks); and late pregnancy (also referred to as the third trimester; 34 ± 3 weeks).

### 2.2. Participants

A total of 141 women who attended the first visit (8–12 weeks) and second visit (24–28 weeks) and successfully participated in a third visit (29–40 weeks) were selected to be included in this secondary analysis. Details of the inclusion and exclusion criteria in the original study are presented in Al-Musharaf et al. [35]. Women were excluded from this analysis if they did not attend the third visit or were not interviewed to complete the sleep questionnaire. Additional exclusion criteria included the presence of systemic or psychiatric disorders; a previous diagnosis by a physician of a sleep disorder, such as sleep apnea syndrome, restless leg syndrome, insomnia, or parasomnia; use of any sleep medication; and involvement in nightshift work. Inclusion criteria included Saudi nationality between 18 and 39 years of age. Based on 80% power and a 95% CI, a sample size of 137 patients was calculated to detect a difference of 1.0 in the PSQI score from the first to third trimester [18].

### 2.3. Data Collection

**Figure 1:**
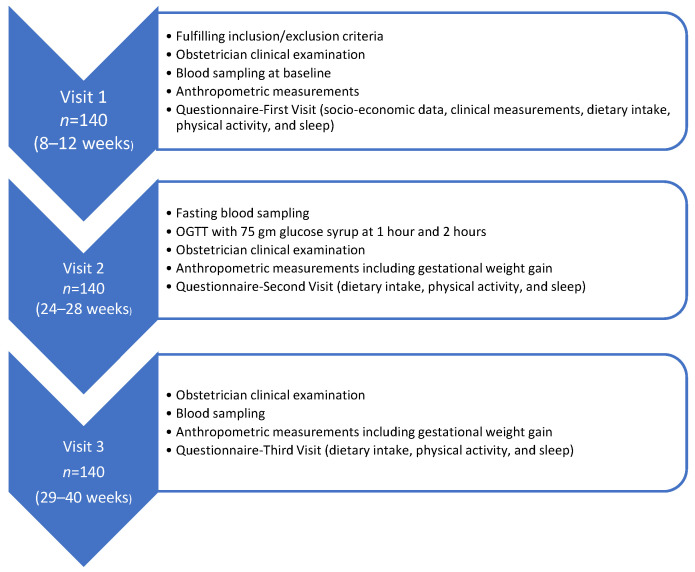
Flow chart of data collection across time.

Vital signs, a medical examination, and anthropometric measurements, including pre-pregnancy BMI, GWG, waist–hip ratio (WHR), and skin-fold thickness, were recorded for all three trimesters [35]. Additionally, data regarding socio-demographics, such as maternal age, employment, monthly income, parity, and past medical and treatment history were collected by a trained interviewer, as well as information from completed International Physical Activity Questionnaires (IPAQs) and a food frequency questionnaire, as detailed previously [35]. Blood samples were obtained from all pregnant women at the three visits for the parent study. The flow chart below describes the data that were collected at all three visits (Figure 1).

#### 2.3.1. Sleep Assessment

The PSQI was used to collect information about sleep quality over the previous month. The validated Arabic version of the questionnaire was used [41]. All the women were interviewed by a trained interviewer to complete the PSQI at all three visits. The PSQI is a subjective sleep-quality questionnaire that contains 19 multiple choice questions to address sleep quality, duration, latency (the time taken to fall asleep), efficiency, disturbances, medications, and daytime sleepiness that interferes with daily activities. These seven component scores are summed to obtain a global PSQI score that reflects the overall sleep quality (range 0–21). Each item receives a score from 0 to 3, and a mean global score of 5 or greater indicates poor sleep quality [42]. Thus, higher scores reflect a poorer quality of sleep [42]. The PSQI has good internal consistency and convergent and divergent reliability in pregnant populations [42]. This study defined short sleep duration with a cut-off of <7 h [9].

#### 2.3.2. Biochemical Assessment

Blood samples were obtained at all three visits. At the first and third visits, the blood samples were collected in a non-fasting state. At the second visit, the women were fasting. The blood samples were immediately transported to the Chair for Biomarkers in Chronic Diseases (CBCD) at King Saud University, where they were processed, aliquoted, and stored at the recommended temperature for further analysis. The lipid profile, including total serum cholesterol (TC); high-density lipoprotein-cholesterol (HDL-C); triglycerides (TGs); HbA1c; and glucose levels were measured at all three visits by a colorimetric method using an automated chemistry analyzer (Konelab, ThermoFisher, Vantaa, Finland), as previously detailed [43]. The intra- and inter-assay coefficients of variation (CV) were TC: 0.7% and 1.5%; HDL-C: 0.6% and 1.2%; TG: 0.9% and 1.8%; glucose: 0.8% and 2.6%, respectively. Low-density lipoprotein cholesterol (LDL-C) was calculated using the Friedewald formula [44]. Dyslipidemia was defined as one or more of the following: TC level > 5.172 mmol/L; low LDL-C level ≥ 3.36 mmol/L; HDL-C level < 1.29 mmol/L; and TG level ≥ 1.7 mmol/L [45]. The HbA1c in whole blood was measured using the DCA Vantage Analyzer (Siemens Healthcare, Erlangen, Germany) point-of-care device.

Participants’ 25-hydroxy vitamin D (25(OH)D) serum levels were measured at all three visits using the ECLIA (Roche Diagnostics GmbA, Mannheim, Germany) and commercially available IDS kits (IDS Ltd., Boldon Colliery, Tyne & Wear, UK). The inter- and intra-assay coefficients of variation (CV) for 25 (OH)D ELISA were 5.3% and 4.6%, respectively, with 100% cross-reactivity to 25(OH) D3 and 75% cross-reactivity to 25(OH) D_2_. The Biomarker Research Program (BRP) laboratory is a contributing body in the vitamin D External Quality Assessment Scheme (DEQAS), and Quality Assurance (QA) standards are retained by ISO 9000 and 17025. The QA department checks the BRP laboratory at consistent times. Participants’ 25(OH)D levels were categorized into deficient (less than 50 nmol/L) or non-deficient (50 nmol/L and greater) groups for a meaningful statistical analysis [46,47].

### 2.4. Statistical Analysis

The data were analyzed using SPSS version 22.0 (IBM Corp, Armonk, NY, USA). Continuous variables are presented as the mean ± standard deviation, while categorical variables are presented as frequencies and percentages. Statistical differences between visits involving continuous variables were analyzed using repeated measures with an ANOVA and a Friedman’s test. Statistical differences between independent groups were analyzed using the independent sample *t*-test and the Mann–Whitney U-test. Statistical differences between visits involving categorical variables were analyzed using the McNemar test. The relationship between sleep scores and other parameters was analyzed using correlation coefficients, as well as linear and logistic regression analyses. A *p*-value of less than 0.05 was considered to be statistically significant.

## 3. Results

### 3.1. General Characteristics of Participants

A total of 141 pregnant women in their first trimester participated in this study. All the participants were married with an average age of 28.0 ± 5.2 (range 18–39) years and a mean BMI of 26.8 ± 6.2 kg/m^2^ at the beginning of pregnancy. Eighty-six of the participants (63.7%) were university graduates or post-graduates, 40 participants (28%) were employed, and 43 participants (30%) were earning more than USD 1300/month. Of the 141, ninety-one (65%) were multipara. The participants’ general characteristics are summarized in Table 1.

### 3.2. Changes in Biochemical and Physical Parameters during Pregnancy

Changes in anthropometric, biochemical, dietary parameters, and physical activity during the course of pregnancy are presented in Table 2. Lipid profiles were significantly elevated from early to late pregnancy (all *p* < 0.001). Although serum vitamin D levels improved significantly in mid and late pregnancy, as compared to the first trimester, most of the patients remained vitamin D-deficient throughout pregnancy (Table 2). The energy intake (percentage of total kcal/day) decreased as pregnancy progressed and was significantly lower in late pregnancy than in early pregnancy (*p* = 0.01).

### 3.3. Percentage and Characterization of Abnormal Sleep Patterns during Pregnancy

**Figure 2:**
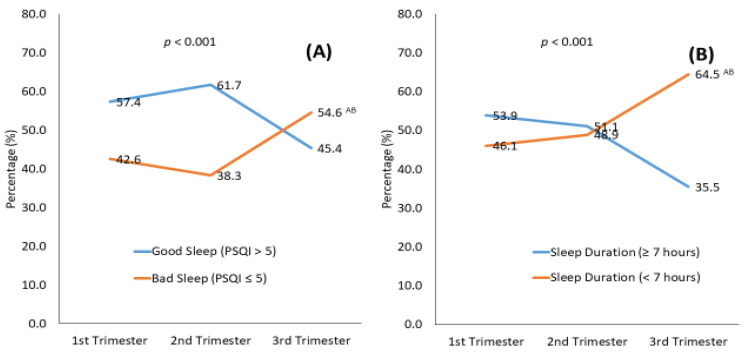
Pittsburgh Sleep Quality Index (PSQI) and score (**A**) and sleep duration (**B**) over the course of pregnancy. A PSQI score of 5 or less indicated poor sleep, and sleep duration less than 7 h indicated a short sleep duration. Superscripts A, B indicate significance from the 1st, 2nd trimester, respectively and AB from both.

The percentage of poor sleep ranged from 38% to 55% and was higher in late pregnancy than in early and mid-pregnancy (*p* = 0.001) (Figure 2). There was an overall poor sleep pattern in the first trimester, which worsened somewhat in the second trimester and then worsened more in the third trimester (Figure 2). The percentage of women reporting short sleep duration ranged from 46% to approximately 65%, with a significant increase from early to late pregnancy. Short sleep duration scores were higher in late pregnancy than in early and mid-pregnancy (*p* < 0.001) (Figure 2).

The mean scores of the individual components of the PSQI at each trimester time point are presented in Table 3. The scores increased progressively as pregnancy advanced for the global PSQI (5.1 ± 2.6, 5.3 ± 2.6, 6.1 ± 2.4; *p* = 0.001); sleep duration (0.8 ± 1.0, 0.9 ± 1.1, 1.3 ± 1.2; *p* = 0.001); and sleep quality (0.7 ± 0.6, 0.9 ± 0.7, 1.2 ± 0.9; *p* < 0.001). Specifically, the total PSQI score was higher in mid and late pregnancy than in early pregnancy (both *p* = 0.001), and the sleep quality score was higher in late pregnancy than in early and mid-pregnancy (both *p* < 0.001).

### 3.4. Factors Predictive of Poor Sleep

#### 3.4.1. Predictors for Sleep in Each Trimester

For each trimester, independent risk factors were associated with poor sleep when using logistic regression (poor sleep, PSQI ≥ 5) as dependent variables against all variables, while adjusting for confounders (not shown in Tables). Multiparity was a significant risk factor for poor sleep in the first trimester (OR, 1.62; CI, 1.11–2.36; *p* = 0.012). Alternatively, higher education was protective against poor sleep in the first trimester (OR, 0.71; CI, 0.51–0.99; *p* = 0.043). All other variables in this study did not show significance, including the biochemical profile and other variables in all three visits.

#### 3.4.2. Predictors for Sleep Changes across Time

- *Vitamin D levels*

**Figure 3:**
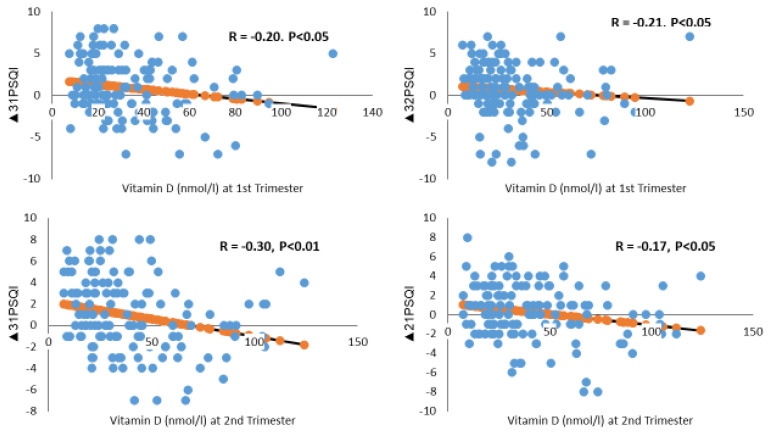
Vitamin D status in relation to changes in Pittsburgh Sleep Quality Index (PSQI) scores across pregnancy. ∆_31_, ∆_21_, and ∆_32_ indicate changes in the PSQI score from the 3rd to the 1st, 2nd to 1st, and 3rd to 2nd trimester.

The above results show that the PSQI index increased significantly throughout pregnancy, indicating that subjects experienced worsening sleep (*p* = 0.001) (Table 3). Specifically, subjects reported a worsening PSQI index in the third trimester, which differed significantly from the PSQI index in both the first and second trimesters (*p* < 0.05 each). These worsening sleeping index scores—∆_31_PSQI and ∆_32_PSQI—were negatively correlated with the second and first trimester vitamin D concentrations (Figure 3). This finding suggests that patients with a higher second-trimester vitamin D concentration experienced fewer sleep problems than those with a lower vitamin D concentration. Further analysis revealed that the PSQI score decreased significantly in the second trimester for subjects with sufficient first-trimester levels of vitamin D (∆_21_PSQI score of −0.9 ± 3.5), compared with subjects who were vitamin D deficient (∆_21_PSQI score of 0.5 ± 2.7) (Table 4). Similarly, the PSQI score decreased significantly in the third trimester for subjects with sufficient second-trimester vitamin D levels (∆_31_PSQI score of −0.9 ± 3.0), compared with subjects who were vitamin D deficient (∆_31_PSQI score of 1.6 ± 3.3).

After adjusting for confounders, a linear regression analysis revealed that a 1 nmol increase in vitamin D concentration in the second trimester was associated with a reduction of 0.2 units in the PSQI score in the third, compared with the first trimester (Table 5).

- *Income*

Income was inversely correlated with changes in the global PSQI and sleep duration scores from the first to the third trimester (r = −0.2, *p* < 0.05) and (r = −0.3, *p* < 0.001), respectively. After adjusting for confounders, a linear regression analysis revealed that low income was predictive of the change in the global PSQI score from the first to the third trimester (Table 5). The significant coefficient of high income indicated that increased income reduced the PSQI index by 0.6 units in the third trimester.

- *Energy Intake*

After adjusting for covariates, a lower energy intake (kcal/day) in the third trimester was associated with the change in global PSQI score from the second to the third trimester (Table 5). A one-unit increase in energy intake in the third trimester was associated with an increase in the PSQI score of 0.15 units.

- *Sitting*

**Figure 4:**
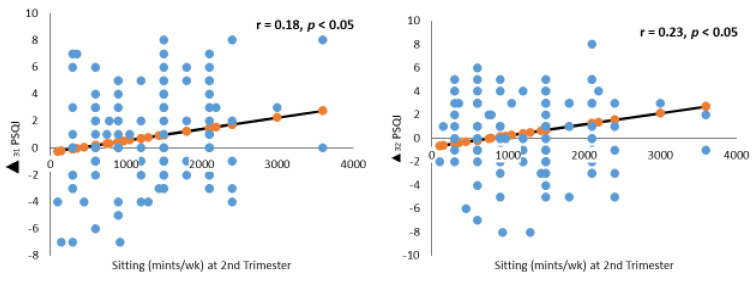
Sitting (minutes/week) in relation to changes in Pittsburgh Sleep Quality Index (PSQI) score across pregnancy. PSQI ∆_31_, ∆_21_, and ∆_32_ indicate changes in the PSQI score from the 3rd to the 1st, 2nd to 1st, and 3rd to 2nd trimester.

In addition, lack of physical activity (sitting) in the second trimester was found to be positively correlated with worsening sleep changes (Figure 4). Both the ∆_31_PSQI and ∆_32_PSQI scores were positively correlated with sitting time in the second trimester, with a correlation coefficient of 0.18 and 0.23, respectively (*p* < 0.05). After adjusting for confounders, a linear regression analysis revealed that an increased sitting time of 1 unit during the second trimester was associated with an increase in the third trimester PSQI score of 0.01 unit (Table 5).

There were no associations between changes in the total PSQI and changes in BMI, WHR, GWG, lipid profile, glucose, and Hba1c. All the variables were incorporated in the regression but did not show significance.

## 4. Discussion

This prospective study reveals a high percentage of poor sleep and short sleep duration throughout pregnancy among pregnant Saudi women. Poor sleep (indicated by a PSQI of 5 or greater) and short sleep duration (less than 7 h per night) ranged from 38% to 55% and 46% to 65%, respectively, with a trend toward a worsening sleep index from early to late pregnancy. The independent predictors of worsening changes in sleep patterns as pregnancy progressed were low income, lower serum vitamin D levels, greater energy intake, and more sitting. Additionally, independent risk factors for poor sleep in the first trimester included multiparity, while possessing university graduate or post-graduate degrees prevented poor sleep.

### 4.1. Alterations in Sleep Patterns

To our knowledge, this is the first study to investigate sleep patterns among a cohort of Saudi women throughout all three trimesters of pregnancy. Our finding that up to 55% of women reported poor sleep, with the highest incidence occurring in the third trimester, is consistent with some but not all previous studies. A recent meta-analysis that included pregnant women (25–30 gestational weeks) from different countries, including the United States, China, and Turkey, reported that 44.5% experienced poor sleep during the second half of pregnancy [11]. Several studies also support our finding that sleep patterns worsen as pregnancy progresses, as reflected by the increasing total PSQI scores over time [9,18,48,49]. A study involving a cohort of 283 Iranian pregnant women found that poor sleep, as assessed by the PSQI, increased steadily from 48% in the first trimester to 63% in the second trimester and 75% in the third trimester [49]. This progressive decrease in sleep quality was further quantified in a recent systematic review of longitudinal studies in which sleep quality decreased by 1.68 points from the second to the third trimester of pregnancy [12]. In contrast to our findings, other studies have reported no significant difference in sleep quality throughout pregnancy [17]. Indeed, Liu et al. reported more disrupted sleep in early and late pregnancy compared with mid-pregnancy, suggesting that the second trimester represented a kind of honeymoon phase [16]. Different conclusions in these studies may, in part, be explained by differences in the population enrolled and the assessment tools. Furthermore, some studies assessed sleep at only two time points during pregnancy, either early, mid, or late pregnancy.

In our cohort of women, short sleep duration (less than 7 h per night) was alarmingly high, reaching 65% in the last trimester of pregnancy. This is almost twice the prevalence that is reported in non-pregnant Saudi women (37%) [50]. Short sleep duration also increased throughout pregnancy which is in line with findings from several other studies [9,16,17]. The incremental increases in poor sleep (both poor quality and short duration) as pregnancy progresses are problematic. Previous studies have demonstrated a relationship between poor sleep and specific poor maternal/fetal/neonatal health outcomes [13,14,15,16,51,52]. Thus, screening for poor sleep in early pregnancy may provide an opportunity to introduce interventions, as the problem only seems to worsen as pregnancy progresses.

### 4.2. Factors Associated with Worsening Sleep Patterns

#### 4.2.1. Demographic Factors and Parity

Few studies have investigated the relationship between demographic factors and poor sleep at different time points over the course of pregnancy, and the findings were inconsistent. Mindell et al. showed that unemployment, low income, and low education were significant predictors of poor sleep during pregnancy [9]. We also found that high income decreased sleep deterioration from early to late pregnancy by 0.6 units in late pregnancy. As birth becomes more imminent, suboptimal sleep environments combined with greater perceived stress and financial strain associated with low income may contribute to worsening sleep in this group of women [53].

Our study also found a higher educational level to be protective against poor sleep during early pregnancy. Having a university or post-graduate degree initially appeared to be protective against poor sleep during the first trimester (OR, 0.71; CI, 0.51–0.99; *p* = 0.043). However, as pregnancy progressed, women with university and post-graduate degrees reported better sleep quality than women without degrees. This finding is similar to Mindell et al. [9] and may, in part, be due to greater health awareness among this group. Although the above-referenced studies support our findings, Colon et al. and Hedman et al. found no association between sleep changes and education or income [20,54]. This may be due to different populations, different sleep, and socio-demographic assessment tools.

Poor sleep quality has been shown to occur with greater frequency in multiparous than in nulliparous women during each trimester of pregnancy [18], and multiparity has also been associated with worsening alterations in sleep patterns throughout pregnancy in some [55], but not all [9,18,20,54], studies. Sleep disturbances in multiparous women may partially be explained by the external demands related to child-bearing and discomfort resulting from possible health complications [56]. In our study, multiparous women reported a modest increase in poor sleep that occurred only in the first trimester, by 1.62 times. This finding differs from other studies that reported poor sleep among multiparous women in the first, second [18], and third trimesters [21] of pregnancy. This discrepancy may be because, as pregnancy progresses, parity-related differences may be obscured by other factors, such as fetal growth and frequent urination, and difficulty finding a comfortable position may considerably disturb sleep, irrespective of parity [9].

#### 4.2.2. Low Vitamin D Levels

Consistent with results from previous studies [57,58], we found that vitamin D levels were deficient at all three time points assessed during pregnancy, though serum levels were higher in mid and late rather than early pregnancy. To the best of our knowledge, this is the first study to assess vitamin D deficiency prospectively and its association with poor sleep patterns in each trimester of pregnancy. We found that pregnant women with lower second-trimester serum vitamin D levels had higher global PSQI scores in late versus early pregnancy. Furthermore, increased vitamin D in the second trimester was associated with a significant reduction in the PSQI score in the third trimester. This suggests that early vitamin D screening could help to identify women that are at risk of developing poor sleep and potentially offer therapeutic interventions to lower the risk.

There are few published studies on the association between poor sleep quality and serum vitamin D levels in the general population [37,59,60,61]. To date, there have been only four studies investigating the correlation between serum vitamin D levels and sleep quality during pregnancy, but the measurements were restricted to only one time point, providing no insight into changes throughout pregnancy [33,38,40]. In a cross-sectional study by Cheng et al. involving 890 Singaporean pregnant women in their second trimester (26–28 weeks), plasma 25(OH)D deficiency was found to be associated with a three-fold increase in the chance of poor sleep quality (PSQI greater than 5), (OR, 3.49; 95% CI, 1.84–6.63) [33]. More recently, Woo et al. assessed 115 African American and Latina pregnant women in their third trimester (29–32 weeks) and found that serum 25(OH)D concentration levels accounted for 17% of sleep quality variance using the PSQI after controlling for race, pre-pregnancy BMI, gestational age, and maternal age [40]. The most recent Turkish study of 153 pregnant women (27–28 weeks) found that poor sleep reached 85.6% (PSQI > 5) among pregnant women with vitamin D deficiency [38]. In contrast, a Turkish study of 91 pregnant women (36 weeks of gestation, third trimester) found no association between plasma 25(OH)D and sleep quality [39]. This discrepancy in the results may be due to a small sample size, limited seasonal variation through the study period, and a high-latitude geographical location.

The mechanism underlying the relationship between poor sleep and serum vitamin D levels is still not clear. One potential mechanism may involve vitamin D hormonal functions and the presence of vitamin D receptors in specific areas of the brain and spinal cord, some of which are thought to play a role in sleep [62,63]. It has been proposed that vitamin D may have direct effects on the initiation and maintenance of sleep by targeting neurons in the diencephalon and other brainstem nuclei, which are linked with circadian clocks [64]. Alternatively, the link between vitamin D deficiency and sleep disturbances may also result from bone disorders that cause non-specific pain [65] and conditions such as non-inflammatory skeletal myopathy [66]. Moreover, studies have proposed that vitamin D deficiency increases inflammation and infection, including those types that interfere with sleep regulation [67].

Some studies have reported that vitamin D supplementation among the general population improves sleep. A randomized clinical trial found that supplementation of 50,000 IU of vitamin D over two months improved sleep duration and quality in people with sleep disorders [68]. Furthermore, vitamin D deficiency among pregnant women results in maternal and fetal complications [13,36]. Consequently, combined with our findings, these results suggest that enhanced vitamin D levels may improve sleep. Additional studies are needed to investigate the role of vitamin D and the potential therapeutic benefit of vitamin D supplementation in improving sleep and potentially reducing poor maternal/fetal/neonatal outcomes.

#### 4.2.3. Energy Intake and Sitting

This study showed that increments in energy intake during the third trimester worsened sleep in the second half of pregnancy by 0.15 units. Short sleep among the general population has been linked to cravings and increased appetite and hunger [69]. Similar to our result, a recent study showed that pregnant obese women with shortened sleep had a greater energy intake in early and late pregnancy [30]. We and others [54,70] found no correlation between BMI or GWG and sleep patterns throughout pregnancy, although recent studies found that excess weight gain from early to late pregnancy was associated with worsening sleep quality [19,30].

Regular physical activity during pregnancy has been shown to improve sleep duration and quality [70,71]. This is consistent with our findings that an increase in sedentary activity, measured by sitting time, was associated with worsening sleep quality in the second half of pregnancy. A recent meta-analysis on different factors concerning sleep showed that poor sleep was associated with less physical activity, with an effect size of 0.13 [70]. However, the authors did not identify any trimester-related associations between sleep disturbance and activity.

The main strength of this study is the prospective design and longitudinal assessment of women across all trimesters of pregnancy. This is also the only study to assess biochemical parameters, in addition to factors that are related to sleep, diet, and physical activity during all three visits. Hence, we adjusted for all potential confounders that may have impacted sleep along with the other variables assessed. Lastly, using trained personnel to carry out the interviews and ensure the completion of the PSQI questionnaire ensured consistency in the follow-up and completeness of the data collection.

There were several limitations to our study. First, we did not assess sleep quality objectively. However, the PSQI has been shown to have good internal consistency and construct validity when used in a pregnant population [9]. Second, the study did not assess other variables that may affect sleep quality, such as frequent urination, nausea, vomiting, difficulty breathing due to increasing abdominal girth, and general physical discomfort, though these factors may be addressed to some extent in the sleep efficiency component of the PSQI. Finally, the questionnaires that were used in this study, including the IPAQ and food frequency questionnaire, may be subject to recall bias.

## 5. Conclusions

In conclusion, poor sleep and short sleep duration are common and poorly recognized sleep disturbances that frequently occur among pregnant women at different time points during their pregnancy. Since poor sleep has been associated with poor maternal, fetal, and neonatal health outcomes, understanding these patterns in different populations, including Middle Eastern women, is crucial. Our results strengthen previously reported correlations between low income, lower education, sitting, and greater energy intake during pregnancy and an increased prevalence of poor sleep. Additionally, we identified a pattern of progressive deterioration in sleep quality as pregnancy advanced. The association between low serum vitamin D levels and worsening sleep quality from early to late pregnancy is an important and unique finding of this study and offers a potential avenue for intervention, both pre-conception and during pregnancy. Additional studies are needed to confirm this association and better elucidate the role that vitamin D deficiency plays in contributing to poor sleep quality during pregnancy and the mechanisms of action underlying the effect. Screening for poor sleep quality and the predictive factors identified in this study could lead to therapeutic interventions to improve maternal and neonatal health outcomes related to abnormal sleep patterns.

## Figures and Tables

**Table 1:** Demographic and clinical characteristics.

Characteristics	Values
Age, years	28.0 ± 5.2
University graduate or higher	86 (63.7)
Employment	40 (28.0)
**Income**	
>1300 USD	43 (30.0)
**Parity**	
Multipara	91 (65.0)
**Pre-pregnancy BMI**	26.8 ± 6.2
Overweight	37 (26.0)
Obesity	29 (21.0)

Values are presented as mean ± SD for continuous variables and *n* (%) for categorical values. United state dollars is represented by USD and Body mass index is represented by BMI. the main parameters subtitles.

**Table 2:** Change in general parameters at the three visits.

	First Trimester	Second Trimester	Third Trimester	*p*-Value
*n*	141	141	141	
Gestational age (weeks)	12.3 ± 3.1	26.0 ± 4.8 ^A^	34.4 ± 3.0 ^AB^	<0.001
**Anthropometric parameters**				
BMI (Kg/m^2^)	27.6 ± 6.0	29.8 ± 6.0 ^A^	30.5 ± 5.3 ^AB^	<0.001
Waist–hip ratio	0.84 ± 0.08	0.93 ± 0.09 ^A^	0.97 ± 0.08 ^AB^	<0.001
Body fat %	35.3 ± 5.2	37.8 ± 2.8 ^A^	38.2 ± 2.5 ^AB^	<0.001
Gestational weight gain (kg)		0.35 ± 0.21	0.35 ± 0.16	0.867
Systolic blood pressure (mmHg)	113.2 ± 12.3	110.6 ± 11.2	110.7 ± 10.9	0.154
Diastolic blood pressure (mmHg)	67.0 ± 8.8	66.7 ± 10.5	68.3 ± 9.1	0.544
**Dietary parameters**				
Energy intake (%)	60.1 ± 12.3	58.5 ± 14.8	51.3 ± 12.1 ^A^	0.010
Carbohydrates intake (%)	90.6 ± 20.7	94.9 ± 30.0	87.5 ± 34.4	0.353
Protein intake (%)	95.7 ± 32.1	89.9 ± 30.9	76.0 ± 31.6	0.093
Fat intake (%)	93.9 ± 28.5	90.3 ± 30.7	77.9 ± 21.9	0.130
Vitamin D intake (IU/day)	149.5 ± 146.4	174.1 ± 167.8	152.3 ± 201.7	0.740
Calcium intake (mg/day)	301.9 ± 380.8	353.4 ± 416.0	262.4 ± 396.2	0.710
Water (mL/day)	1407.8 ± 743.5	1422.9 ± 691.8	1562.5 ± 786.9	0.735
Tea (mL/day)	533.3 ± 696.4	187.3 ± 110.1 ^A^	177.0 ± 90.4 ^A^	0.005
Coffee (mL/day)	78.9 ± 65.7	78.9 ± 75.5	121.3 ± 80.0	0.161
**Physical activity**				
Sitting (min/wk)	1137.0 ± 728.0	1296.0 ± 721.0	1311.0 ± 601.0	0.440
Low physical activity (min/wk)	351.0 ± 474.0	532.0 ± 660.0	109.0 ± 62.0	0.140
**Biochemical parameters**				
Calcium (mmol/L)	2.1 ± 0.2	2.1 ± 0.2	2.1 ± 0.3	0.174
Total cholesterol (mmol/L)	5.2 ± 1.1	6.6 ± 1.5 ^A^	6.6 ± 1.3 ^A^	< 0.001
HDL-cholesterol (mmol/L)	1.3 ± 0.4	1.6 ± 0.5 ^A^	1.4 ± 0.5 ^A^	< 0.001
LDL-cholesterol (mmol/L)	3.2 ± 0.8	4.1 ± 1.2 ^A^	4.1 ± 1.1 ^A^	< 0.001
Glucose (mmol/L)	4.9 ± 1.1	4.8 ± 1.0	5.1 ± 1.5 ^B^	0.020
Triglycerides (mmol/L)	1.4 ± 0.5	2.1 ± 0.8 ^A^	2.4 ± 1.0 ^AB^	< 0.001
Vitamin D (nmol/L)	32.9 ± 20.2	40.2 ± 25.6 ^A^	38.3 ± 22.9 ^A^	< 0.001
HbA1c	5.1 ± 0.5	4.8 ± 0.5 ^A^	5.1 ± 0.6 ^B^	< 0.001

Data presented as mean ± SD for continuous variables and *n* (%) for categorical variables; *p*-values obtained from repeated measures with ANOVA and Friedman’s tests. Superscripts A, B indicate significance from the 1st, 2nd trimester, respectively. Body mass index is represented by BMI, glycated hemoglobin by HbA1c. Bold font represents the main parameters subtitles.

**Table 3:** Mean PSQI scores at each trimester visit.

	First Trimester	Second Trimester	Third Trimester	*p*-Value
***n***	141	141	141	
**Week of gestation**	12.3 ± 3.1	26.0 ± 4.8 ^A^	34.4 ± 3.0 ^AB^	<0.001
**Sleep components**				
Habitual sleep efficiency	1.3 ± 1.4	1.2 ± 1.3	1.5 ± 1.3	0.104
Sleep duration	0.8 ± 1.0	0.9 ± 1.1	1.3 ± 1.2 ^A^	0.001
Sleep latency	1.0 ± 1.1	1.1 ± 1.1	1.1 ± 1.1	0.514
Sleep disturbance	0.8 ± 0.7	0.9 ± 0.7	0.7 ± 0.7	0.064
Sleep quality	0.7 ± 0.6	0.9 ± 0.7	1.2 ± 0.9 ^AB^	<0.001
Sleep medication	0.0 ± 0.2	0.0 ± 0.0	0.1 ± 0.2	0.532
Day dysfunction	0.4 ± 0.7	0.3 ± 0.6	0.3 ± 0.6	0.465
Fall asleep (in minutes)	51.9 ± 41.6	30.7 ± 27.0 ^A^	38.0 ± 31.0 ^A^	<0.001
Total sleep hours (hours/day)	7.9 ± 2.8	7.1 ± 2.5 ^A^	8.8 ± 4.9 ^B^	<0.001
**Total PSQI score**	5.1 ± 2.6	5.3 ± 2.6 ^B^	6.1 ± 2.4 ^A^	0.001

Data presented as mean ± SD for continuous variables; *p*-values for continuous variables are obtained from repeated measures with ANOVA and Friedman’s tests. Pittsburgh Sleep Quality Index (PSQI). Superscripts A, B indicate significance from the 1st, 2nd trimester, respectively and AB from both. Bold font represents the subtitles.

**Table 4:** Vitamin D and changes in sleep index.

Change in PSQI	Vitamin D Status in 1st Trimester			Vitamin D Status in 2nd Trimester		
	Sufficient	Deficient	*p*-Value	Sufficient	Deficient	*p*-Value
∆_31_PSQI	−0.3 ± 3.8	1.2 ± 3.3	0.060	−0.9 ± 3.0	1.6 ± 3.3	<0.001
∆_21_PSQI	−0.9 ± 3.5	0.5 ± 2.7	0.034	−0.8 ± 3.2	0.7 ± 2.6	0.008
∆_32_PSQI	0.6 ± 3.2	0.7 ± 3.1	0.926	−0.1 ± 3.3	0.9 ± 3.0	0.113

∆_31_, ∆_21_, and ∆_32_ indicate changes in the PSQI score from the 3rd to the 1st, the 2nd to the 1st, and the 3rd to the 2nd trimester.

**Table 5:** Predictors of poor sleep (total PSQI) changes during pregnancy.

	∆_31_PSQI		∆_21_PSQI		∆_32_PSQI	
	B ± SE	*p*-Value	B ± SE	*p*-Value	B ± SE	*p*-Value
High income	−0.60 ± 0.26	**0.025**	−0.11 ± 0.22	0.611	−0.49 ± 0.25	0.054
**First trimester**						
NS						
**Second trimester**						
Vitamin D (nmol/L) #	−0.20 ± 0.01	**0.039**	−0.01 ± 0.01	0.621	−0.03 ± 0.02	0.107
Sitting (in min/wk) #	0.00 ± 0.00	0.216	0.00 ± 0.00	0.373	0.01 ± 0.00	**0.044**
**Third trimester**						
Energy intake (%) Kcal/day #	0.01 ± 0.04	0.771	−0.03 ± 0.05	0.589	0.15 ± 0.07	**0.048**

Data are Beta ± standard error obtained from linear regression with # indicating adjustment for age, BMI, parity, income, lipid levels, diet, and physical activity at the respective visits. PSQI ∆_31_, ∆_21_, and ∆_32_ indicate changes in the PSQI score from the 3rd to the 1st, 2nd to 1st, and 3rd to 2nd trimester. Not significant indicated by NS; *p*-value < 0.05 considered significant.

## Data Availability

Not applicable.
